# Structural equation modeling for the effects of family dysfunctions and communication on perceived mental health status among under/graduate students in the U.S.

**DOI:** 10.1371/journal.pone.0301914

**Published:** 2024-04-24

**Authors:** En-Jung Shon, Lena Lee

**Affiliations:** 1 Department of Social Welfare, Duksung Women’s University, Seoul, South Korea; 2 Department of Teaching, Curriculum, and Educational Inquiry, Miami University, Oxford, Ohio, United States of America; St John’s University, UNITED STATES

## Abstract

**Background:**

Many of the college students expressed experiencing moderate or severe psychological distress. In their emergence as adults, college students encounter significant periods of drastic change. Family functions play a crucial role in determining individuals’ mental health status.

**Objectives:**

The first objective of this study was to investigate whether family communication mediates the relationships between family dysfunctions and perceived mental health status among under/graduate students. The second objective was to investigate whether the mediation effects of family communication on the relationships between family dysfunctions and perceived mental health status differ by gender.

**Methods:**

The current study applied Minuchin’s Structural Family Theory with under/graduate students (*N* = 348) in Ohio. Structural Equation Modeling was conducted to examine the effects of family dysfunctions and communication on perceived mental health status among under/graduate students.

**Results:**

Disengaged family function significantly influenced family communication, which, in turn, significantly impacted perceived mental health (mediation effect). Enmeshed family function did not show a significant relationship with family communication, whereas family communication had a significant impact on perceived mental health (no mediation effect). The model comparison test indicated that there were differences between males and females: the structural weights of the current path models were consistent with the full models, and there was a significant difference in the effect of parents’ marital status on perceived mental health, with males being significantly impacted in both models.

**Conclusions:**

Practitioners should recognize that improving positive communication skills within the family unit can be a realistic approach to supporting the perceived mental health of under/graduate students. Colleges/universities should offer regular education programs to promote an understanding of the relationship between family communication strategies and the mental health of under/graduate students.

## Introduction

### Under/graduate students’ mental health

The Healthy Minds Study gathered information from 373 campuses across the country, revealing that during the 2020–2021 academic year, more than 60% of college students met the criteria for at least one mental health problem [1]. The National College Health Assessment: Nationwide survey [2] reported that nearly 75% of students expressed experiencing moderate or severe psychological distress. In their emergence as adults, college students encounter significant periods of drastic change. The onset of newfound independence during the period of emerging adulthood presents difficulties, frequently resulting in higher levels of perceived stress, engagement in behaviors with risks, elevated rates of suicide, and an increase in the utilization of mental health resources [3, 4]. Mental health problems in college are linked to lower academic achievement and a higher risk of dropping out or not completing college [5]. Without adequate support, individuals face challenges in their academic performance, may withdraw from courses, experience decreased socio-emotional well-being [5, 6], and have an increased likelihood of developing suicidal thoughts and attempting suicide [7, 8]. Consequently, intervention and support during this critical period can significantly impact on overall mental well-being and public health outcomes.

### Family dysfunctions, communication, and students’ mental health

While many students move away from home to attend university, the relationships they maintain with their family members continue to significantly impact their mental health [9]. It is well known that family functioning and the mental health of young adults, such as depressive symptoms, are significantly interconnected [10]. Family dysfunctions (such as unbalanced family boundaries like disengagement or enmeshment) can lead to a negative family climate, which in turn has a detrimental impact on children’s mental health status [11–13]. In general, family dysfunction indicates irregularities in the family system’s differentiation and adaptability, which could cause family malfunctioning, such as inadequate task performance [14] Olson (2011) [15] who proposed the Circumplex Model noted that families with balanced cohesion enable their members to be independent while maintaining appropriate connections. In contrast, families with unbalanced cohesion (such as being disengaged or enmeshed) display either extreme emotional dependency and over involvement or very limited involvement among family members.

Families that are disengaged have strong limits between their members and show minimal emotional bonds and interactions among them [15, 16]. Wark et al. (2003) [17] assumed that increased levels of family disengagement could be connected to emotional neglect as a type of child maltreatment; and hypothesized that emotional child neglect would be related to lower levels of balanced cohesion and adaptability and would be associated with higher levels of psychological distress. Findings, including 69 female and 22 male undergraduate students, indicated that individuals who experienced emotional neglect from their family members reported higher levels of psychological distress compared to their counterparts. Young et al. (2011) [18] discussed how children’s perceptions of being emotionally ignored by their parents were associated with the possibility of having mental health issues as they grow older. Findings showed that children who perceived their parents’ emotional neglect reported higher levels of psychiatric disorders (anxiety and mood disorders). Coll et al. (2008) [19] investigated the relationships between family engagement and children’s behavioral disorders, reporting significant differences in conduct-disordered behaviors between children with lower levels of family engagement and their counterparts. Other prior studies [20–22] have also found that perceived attachment security from parents is significantly associated with college students’ academic, social, and overall adjustment. These findings strengthen the viewpoint that perceived secure attachment, which is different from disengagement, aids adolescents in effectively navigating stressful situations.

Craddock et al. (2009) [23] explicated how encountering diverse parenting approaches and family dynamics can predict the degrees of dysfunctional and functional perfectionism in young adults (264 university students) during their initial year at university. The findings indicated that family enmeshment significantly predicted dysfunctional perfectionism. The authors commented that since dysfunctional perfectionism refers to a psychological tendency or mindset in which an individual sets unrealistically high standards for themselves and engages in rigid and overly critical self-evaluation, it could lead to negative psychological outcomes. Segrin, Wozidlo, Givertz, Bauer, and Murphy (2012) [24] targeted 538 parent and young adult dyads and hypothesized that dysfunctional family processes, such as over-parenting, are associated with negative child outcomes. Findings indicated that over-parenting is linked to poor parent-child communication and has an indirect effect on lower family satisfaction. Schiffrin et al. (2014) [25] found that college students (n = 297) who were exposed to over-control from their parents reported higher levels of depression and lower levels of life satisfaction. Students also argued that their basic psychological needs for autonomy and competence were severely disturbed by too intense relationships and over-parenting from their family members. These prior study findings acknowledge that excessive involvement in relationships or overly controlling parenting can lead to a state of enmeshment, where emotional bonds become overly intense and family boundaries become unclear, potentially having negative effects on the family’s well-being [26]. These previous studies have proposed that family dysfunctions, such as disengagement and enmeshment, have a significant impact on the psychological well-being of college students (emerging adults–young adults). Functional families are cohesive but not enmeshed. Families that uphold suitable boundaries within the family unit tend to sustain favorable communication patterns and experience higher levels of family satisfaction [25].

In addition, previous studies [27, 28] have shown that inadequate family communication was significantly associated with behavioral problems in adolescents and young adults. Moreover, the quality of family communication was found to be more important on children’s life satisfaction than financial resources [27]. These findings indicate that improved communication can enhance family members’ self-esteem and alleviate feelings of loneliness. As family units maintain a higher quality of communication, each member feels closer, valued, and better understood by others [29]; consequently, individuals can maintain higher levels of self-esteem [30].

### Minuchin’s structural family theory

Structural family theory was initiated by Salvador Minuchin in the 1960s and has continued to evolve through his ongoing practice [31]. The focus of the theory is on ’family structure,’ and its concepts are based on the invisible and unspoken rules that govern family members’ interactions. Minuchin and his colleagues argued that positive emotional well-being and behaviors among family members are linked to appropriate authority, rules, subsystems, boundaries, and adequate family communication [32].

The structural family theory emphasizes that while family members are connected to the family unit, each member should maintain a degree of physical and emotional separation to ensure their effective functioning. Frequently, the boundaries of family units can be either rigid (such as physical or emotional disengagement) or fluid (as seen in enmeshment, where members maintain overly close boundaries, inhibiting privacy and separateness) [31, 32]. According to Madden-Derdich, Estrada, Updegraff, and Leonard (2002) [33], establishing appropriate boundaries between parents and children influences the development of young adolescents and enhances their ability to form and maintain alliances and communication skills. Furthermore, there is a strong emphasis on the fact that external boundaries, like the separation of the family unit from outside systems, should not disturb the private matters of family members [32].

Functional family communication is established through verbal and nonverbal consistency, as well as by allowing for family rules that enable all members to discuss specific issues, share suggestions, and express their thoughts without experiencing any pressure. Unclear, unbalanced communication, or a lack of communication could lead to family malfunctions or problems [32]. Minuchin and his colleagues [31] emphasized that improved family communication skills could promote other positive family dynamics or mitigate negative family issues.

### Gender differences

Minuchin’s [31] structural family theory also emphasized that an understanding of family structure and functions should take into account cultural variability. Different cultural backgrounds can lead to variations in the formation of family structures, including differences in boundaries, communication styles, and family hierarchy and power structure [34]. Gender differences in individual functions could be directly or indirectly linked to diverse cultural backgrounds. Generally, gender defines the characteristics that a society or culture delineates as masculine or feminine [35]. Scholars have noted that gender role expectations and cultural norms lead females and males to behave and communicate differently [36]. Typically, females tend to favor interpersonal orientation and attentiveness to others’ emotional status, while males often focus more on task-oriented interactions centered on the problem-solving process. Different characteristics of the two groups might be directly or indirectly related to social behavior differences and further different functions or reactions in family units [37]. The distinct characteristics of these two groups could be directly or indirectly linked to variations in social behavior, leading to diverse functions and reactions within family units [38].

However, prior studies have reported inconsistent findings regarding gender differences in family functions and psychological well-being: while some studies indicated gender differences, others did not. Telzer and Fuligni (2013) [39] investigated the effects of positive and negative family relationships on gender differences in psychological distress targeting 681 adolescents; and the findings indicated that females are more likely to be impacted by negative relationships than males. The gender differences were eliminated when family members maintained higher levels of family cohesion (i.e., lower levels of disengagement). The extent of gender differences was reduced when family members engaged in more frequent positive interactions. Guassi Moreira and Telzer (2015) [40] examined how family cohesion impacts depressive symptoms in emerging adulthood among 338 college students. The findings indicated that individuals with higher levels of family cohesion reported lower levels of these symptoms compared to their counterparts. Regarding gender differences, a significant effect of family cohesion on depressive symptoms was observed within the female group.

With regard to the effects of family communication, Levin et al. (2012) [27] assessed whether both male and female groups were significantly influenced by family communication in their life satisfaction and found that there were no significant gender differences. However, Proctor et al. (2009) [30] found that effect of family communication patterns on life were differently influenced for the two groups.

Research continuously reports inconsistent findings regarding the relationships between family functioning, communication, and psychological well-being in different gender groups. Therefore, the current study still needs to investigate whether significant gender differences will exist in the relationships among family dysfunctions, communication, and perceived mental health status.

### Theoretical framework of the current study

**Fig 1:**
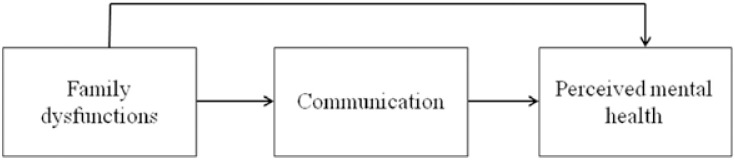
Research design for the current study.

Minuchin’s Structural Family Theory and prior studies propose that families exhibiting negative dynamics (i.e., family dysfunctions such as disengagement or enmeshment) in their structures have fewer opportunities to effectively communicate and cope with stressors, thereby negatively impacting mental health status. This highlights the importance of mediating effect of family communication on the relationship between family dysfunctions and perceived mental health status. Additionally, with regard to Minuchin’s Structural Family Theory, it’s crucial to take into account cultural diversity to comprehend both the structure and functions of families. Gender stands out as among the most significant cultural variables in this regard [32] (Fig 1).

### Objectives of the study

Very few studies have investigated the influence of particular family dysfunctions, such as disengaged and enmeshed statuses, on communication dynamics and their subsequent impact on the perceived mental health status of undergraduate and graduate students. Notably, there is limited information available on how specified family dysfunctions affect family communication patterns. Furthermore, prior studies indicate inconsistent findings regarding gender differences in family structure and communication patterns. Consequently, the aim of the present study is to comprehend the relationships among family dysfunctions, communication patterns, and the mental health status of undergraduate and graduate students, and to investigate whether there are gender differences in these relationships. To reflect the study objectives, the details of the hypotheses are as follows:

1. Family communication will mediate the relationships between family dysfunctions such as disengaged status and enmeshed status and perceived mental health status among under/graduate students.
2. The mediation effects of family communication on the relationships between family dysfunctions such as disengaged status and enmeshed status and perceived mental health status will differ by gender.

## Materials and methods

### Data collection procedure

In this study, data were gathered using a convenient sampling technique, focusing on undergraduate/graduate students from two universities and multiple community churches in Ohio, U.S. The goal was to improve the applicability of the findings to college students in Ohio. By including participants from both universities and community churches, this study sought to enhance its generalizability to college or university students in Ohio. Approval for the data collection process was obtained from the Institutional Review Board of the lead university. From September 2019 to March 2020, a web-based survey that participants completed independently was carried out. After obtaining approval from the Institutional Review Board (IRB, Approval #03204e), the research team’s principal investigator (PI) managed the recruitment process through e-mails obtained from universities and community churches. Student e-mails for data collection were provided by the Institutional Research and Effectiveness of the lead universities. The designated gatekeeper of the community churches collected e-mails from potential survey participants and delivered them to the research team’s principal investigator (PI). The PI had the responsibility of supervising the web-based data collection procedures. Through the web-based survey, potential participants were encouraged to review the survey guidelines and consent form. Once they agreed to the consent form, they were automatically directed to the main questionnaires. The agreement is a written statement. If the target groups want to participate in the survey, they should click the ‘agree’ button. When they click the agree button, the system automatically assigns a ‘random ID number (anonymous ID)’, and the research team members cannot figure out who they are.

The survey included socioeconomic characteristics (age, gender, race, family income, marital status of parents, the school year of the participants, parents [mother and father] education attainment), family dysfunctions (disengagement and enmeshed), family communication, and psychological distress. A total of 348 individuals were included in the ‘complete’ data.

### Measures

For socioeconomic characteristics, race was recoded into four categories such as Whites (reference variable for the analysis), African Americans, Hispanics, and Asians. Gender was originally coded as four categories including male, female, other, or do not want to indicate. In the complete data, no one responded for ‘other’ or ‘do not want to indicate,’ so it was recoded as binary, with male = 1 and female = 0. Regarding marital status of parents, married (coded as 1) and unmarried (coded as 0) statuses were included. The school year was coded on a scale of 1 (1st-year undergraduate) to 6 (graduate student), and the reported range was 1 through 6 (skewness = |0.56|). Parents’ education level was coded on a scale of 0 (no formal education) to 8 (doctorate or higher), and the reported range was also 0 through 8 (mother’s skewness = |0.82|, father’s skewness = |0.80|). Family household income was originally coded as an open-end question, and recoded based on skewness and kurtosis into three categories: 1 = $0 to $44,999, 2 = $45,000 to $139,999, and 3 = $140,000 to $5,000,000).

The representative scale evaluating family functions is the Family Adaptability and Cohesion Evaluation Scale (FACES) IV [15]. Olson (2011) [15] suggested balanced cohesion, disengaged, and enmeshed statuses to evaluate family functions, especially for family cohesion. The balanced cohesion is considered a balanced scale, while the remaining concepts (disengaged and enmeshed) are considered unbalanced scales. The current study employed two unbalanced scales to examine the status of family dysfunctions. The definition of disengaged status is the exhibition of inflexible boundaries, which are evident in the cold, indifferent, unsupportive, and emotionally withdrawn familial connections [41]. The disengaged scale includes the seven items: (1) we get along better with people outside our family than inside (2) family members seem to avoid contact with each other when at home (3) family members know very little about the friends of other family member (4) family members are on their own when there is a problem to be solved (5) our family seldom does things together (6) family members seldom depend on each other (7) family members mainly operate independently. Response options ranged from 1 to 5 (1 = strongly disagree ~ 5 = strongly agree), and the observed range of the summed score was from 1 to 35 (Cronbach’s alpha = 0.86; scale *M* and *SD* = 15.52, 5.79 respectively). A higher summed score suggests weaker family functioning concerning the disengaged status.

The boundaries in enmeshed families are blurred, resulting in confusion of roles and expectations, excessive and inappropriate reliance of parents on their children for support, and children becoming emotionally dependent and reluctant to be separated from their family members [42]. The enmeshed scale also includes seven items: (1) we spend too much time together (2) family members feel pressured to spend most free time together (3) family members are too dependent on each other (4) family members have little need for friends outside the family (5) we feel too connected to each other (6) we resent family members doing things outside the family (7) family members feel guilty if they want to spend time away from the family. Response options for the both scales have five categories (1 = strongly disagree ~ 5 = strongly agree). Response options ranged from 1 to 5, and the observed range of the summed score was from 1 to 35 (Cronbach’s alpha = 0.76; scale *M* and *SD* = 14.58, 4.67 respectively). A higher summed score suggests weaker family functioning concerning the enmeshed status.

Olson (2011) [15] also suggested family communication status as one of the most important measures to understand family functions. Family communication incorporates both verbal and non-verbal reactions and differs from interpersonal or intergroup communication with other members (friends, co-workers, neighbors, etc.). It could help individuals develop coping and resilience strategies to manage family and psychological distress [43]. For communication status, 10 items are included: (1) Family members are satisfied with how they communicate with each other (2) Family members are very good listeners (3) Family members express affection to each other (4) Family members are able to ask each other for what they want (5) Family members can calmly discuss problems with each other (6) Family members discuss their ideas and beliefs with each other (7) When family members ask questions of each other, they get honest answers (8) Family members try to understand each other’s feelings (9) When angry, family members seldom say negative things about each other (10) Family members express their true feelings to each other. Response options for the scale has five categories (1 = strongly disagree ~ 5 = strongly agree). Observed range of the summed score was from 10 to 50 (Cronbach’s alpha = 0.92; scale *M* and *SD* = 37.09, 7.84 respectively). A higher summed score suggests better family functioning concerning the family communication status.

Psychological distress in this study was measured by using the question, “how would you describe your mental health in general (1 = poor, fair, good, very good, or 5 = excellent)?” As quasi-interval scale, observed average score was 3.25 (*SD* = 1.10, range = 1–5). A higher level of score suggests better perceived mental health status. For more detailed information, Tables 1 and 2 provide additional details.

**Table 1:** Sample characteristics on socio-demographics and family functions.

		female		male		chi-square or t
		*N*	*%*	*N*	*%*	
Race	Whites	95	37.10	37	40.20	3.317
	African Americans	41	16.00	8	8.70	
	Hispanics	49	19.10	17	18.50	
	Asians	71	27.70	30	32.60	
Parents marital status	Unmarried	70	27.30	23	25.00	0.19
	Married	186	72.70	69	75.00	
Income	$0 to $44,999	64	25.00	20	21.70	0.653
	$45,000 to $139,999	108	42.20	38	41.30	
	$140,000 to $5,000,000	84	32.80	34	37.00	
		*M*	*SD*	*M*	*SD*	
Age		21.57	4.94	22.05	5.20	-0.801
School year		3.3	1.50	3.3	1.53	-0.041
Mother’s education		6.44	1.68	6.55	1.62	-0.578
Father’s education		7.59	1.81	7.71	1.89	-0.542
Family functions	Disengaged status	15.27	5.70	15.92	6.01	-0.937
	Enmeshed status	14.39	4.56	14.87	5.08	-0.831
	Communication	36.61	8.11	36.38	7.05	-1.854

* p < .05, ** p < .01, *** p < .001

**Table 2:** Scale descriptive characteristics: Disengaged, enmeshed, and family communication.

	**Disengaged Status Questionnaires**	**Female**		**Male**	
		***M***	***SD***	***M***	***SD***
Item 1	We get along better with people outside our family than inside	2.71	1.16	2.86	1.21
Item 2	Family members seem to avoid contact with each other when at home	1.90	1.00	1.86	1.03
Item 3	Family members know very little about the friends of other family member	2.17	1.11	2.22	1.17
Item 4	Family members are on their own when there is a problem to be solved	1.84	1.05	1.91	1.03
Item 5	Our family seldom does things together	2.07	1.16	2.09	1.12
Item 6	Family members seldom depend on each other	2.10	1.12	2.15	1.16
Item 7	Family members mainly operate independently	2.66	1.25	2.86	1.20
	**Enmeshed Status Questionnaires**	***M***	***SD***	***M***	***SD***
Item 1	We spend too much time together	2.20	0.98	2.20	0.98
Item 2	Family members feel pressured to spend most free time together	2.20	1.02	2.20	1.02
Item 3	Family members are too dependent on each other	2.13	0.97	2.13	0.97
Item 4	Family members have little need for friends outside the family	1.96	0.99	1.96	0.99
Item 5	We feel too connected to each other	2.38	1.19	2.38	1.19
Item 6	We resent family members doing things outside the family	1.50	0.85	1.50	0.85
Item 7	Family members feel guilty if they want to spend time away from the family	2.07	1.14	2.07	1.14
	**Family Communication Questionnaires**	***M***	***SD***	***M***	***SD***
Item 1	Family members are satisfied with how they communicate with each other	3.51	1.04	3.84	0.84
Item 2	Family members are very good listeners	3.57	1.11	3.77	1.01
Item 3	Family members express affection to each other	3.79	1.12	3.88	0.99
Item 4	Family members are able to ask each other for what they want	3.97	0.97	4.02	0.86
Item 5	Family members can calmly discuss problems with each other	3.46	1.16	3.73	1.07
Item 6	Family members discuss their ideas and beliefs with each other	4.01	0.96	4.08	0.92
Item 7	When family members ask questions of each other, they get honest answers	3.91	1.02	4.20	0.79
Item 8	Family members try to understand each other’s feelings	3.84	1.06	3.96	0.91
Item 9	When angry, family members seldom say negative things about each other	2.91	1.26	3.04	1.20
Item 10	Family members express their true feelings to each other	3.80	0.99	3.87	0.92

### Statistical analyses

Univariate frequencies, descriptive statistics, histograms, and bivariate scatter plots were examined for outliers, adequate variability, skewness (< 2) and kurtosis (< 21) [44]. There was no violation on items of the scales. Moreover, non-collinearity (Pearson’s r < .08) and non-problematic multivariate outliers (i.e., cases with nonextreme discrepancies across the squared Mahalanobis distance scores) were expected for the structural equation modeling (SEM) analyses [45, 46]. Bivariate correlation reported non-multicollinearity across the measures.

The Mahalanobis distance scores indicated that the complete data did not show extreme outliers. The current study employed chi-square tests to examine the associations between variables and conducted independent sample t-tests to assess the differences in means for variables based on gender.

For the structural equation modeling, confirmatory factor analyses for disengaged status, enmeshed status, and communication status were initially performed. (1) Regarding hypothesis 1: unconstrained full structural equation modeling analyses were applied to assess the mediation effects of family communication on the relationship between family dysfunctions (disengaged or enmeshed status) and perceived mental health status among under/graduate students. (2) Then, for the hypothesis 2, the current study conducted multiple group structural equation modeling analyses based on gender (male vs. female). These analyses aimed to investigate whether the mediation effect of family communication on the relationship between family dysfunctions and perceived mental health status would vary across genders or not [45, 47]. The Sobel test was applied to evaluate the mediation effects of family communication on the relationships between family dysfunctions and perceived mental health status. The Bootstrap function in the Amos program was used to investigate direct and indirect effects for the model in the current study.

The structural equation modeling fit was evaluated based on the goodness-of-fit indices such as chi-square/degree of freedom (≤5), Comparative Fit Index (CFI; acceptable fit ≥ 0.09), Root Mean Square Error of Approximation (RMSEA; acceptable fit ≤0.05), and Standardized Root Mean Square Residual (SRMR). SRMR value close to zero indicates an acceptable fit. Chi-square/df, CFI, RMSEA, and SRMR are well-known in the context of factor-based structural equation modeling, where cutoff values have long been established [48–50].

## Results

### Sample characteristics

The final sample included 348 under/graduate students. The mean age of the participants was 21.69 years, with 73.6% being female and 26.4% being male. Table 1 presents sample characteristics by gender. There was no significant difference in socio-demographic factors and family functions between males and females. The tendencies within male or female groups were similar. Whites showed the highest percentage among the four racial groups, followed by Asians, Hispanics, and Africans in that order. The majority of the participants reported that their parents maintained a married status. Many of them were included in the $45,000 to $139,999 range group. The average age was 21.57 for females and 22.05 for males. The average school year was 3.3 for both groups. Both groups’ fathers reported higher educational attainment than their mothers. As mentioned earlier, each family function itself was not significantly different between genders.

Table 2 presents descriptive information regarding the scales for the constructs of disengaged status, enmeshed status, and family communication. Each construct consists of 7 to 10 items, with corresponding average scores and standardized deviation scores.

### Hypotheses evaluation

The confirmatory factor analyses for family dysfunctions (disengaged and enmeshed) and family communication showed good model fits. For disengaged status, *X*^2^/df = 1.392, CFI = 0.997, RMSEA = 0.035; and for enmeshed status, *X*^2^/df = 2.107, CFI = 0.989, RMSEA = 0.057. Regarding family communication, *X*^2^/df = 1.864, CFI = 0.936, RMSEA = 0.051. All items for disengaged, enmeshed, and communication status significantly reflected their constructs.

Regarding hypothesis 1, unconstrained mediation model was tested. The model’s fit scores regarding the relationships among disengaged function, family communication, and perceived mental health were as follows: *X*^2^/df = 1.864, CFI = 0.936, RMSEA = 0.051, SRMR = 0.07. Disengaged family function significantly influenced family communication (standardized B = -0.717, *p* < .001), which, in turn, significantly impacted perceived mental health (standardized B = 0.408, *p* < .001; Sobel test = -3.94, *p* < .001). The indirect impact of disengaged family function on perceived mental health, mediated by family communication, resulted in a negative effect size of -0.293. Disengaged family function did not show a significant direct impact on perceived mental health (standardized B = 0.118, *p* = 0.208).

The model’s fit scores regarding the relationships among enmeshed function (unconstrained full model), family communication, and perceived mental health were as follows: *X*^2^/df = 1.675, CFI = 0.943, RMSEA = 0.045, SRMR = 0.07. Enmeshed family function did not show a significant relationship with family communication, whereas family communication had a significant impact on perceived mental health status (standardized B = 0.333, *p* < .001; Sobel test = -1.178, *p* = 0.238). The indirect impact of enmeshed function on perceived mental health through family communication resulted in a value of -0.026. Enmeshed function did not report a significant direct impact on perceived mental health (standardized B = 0.118, *p* = 0.208).

**Fig 2:**
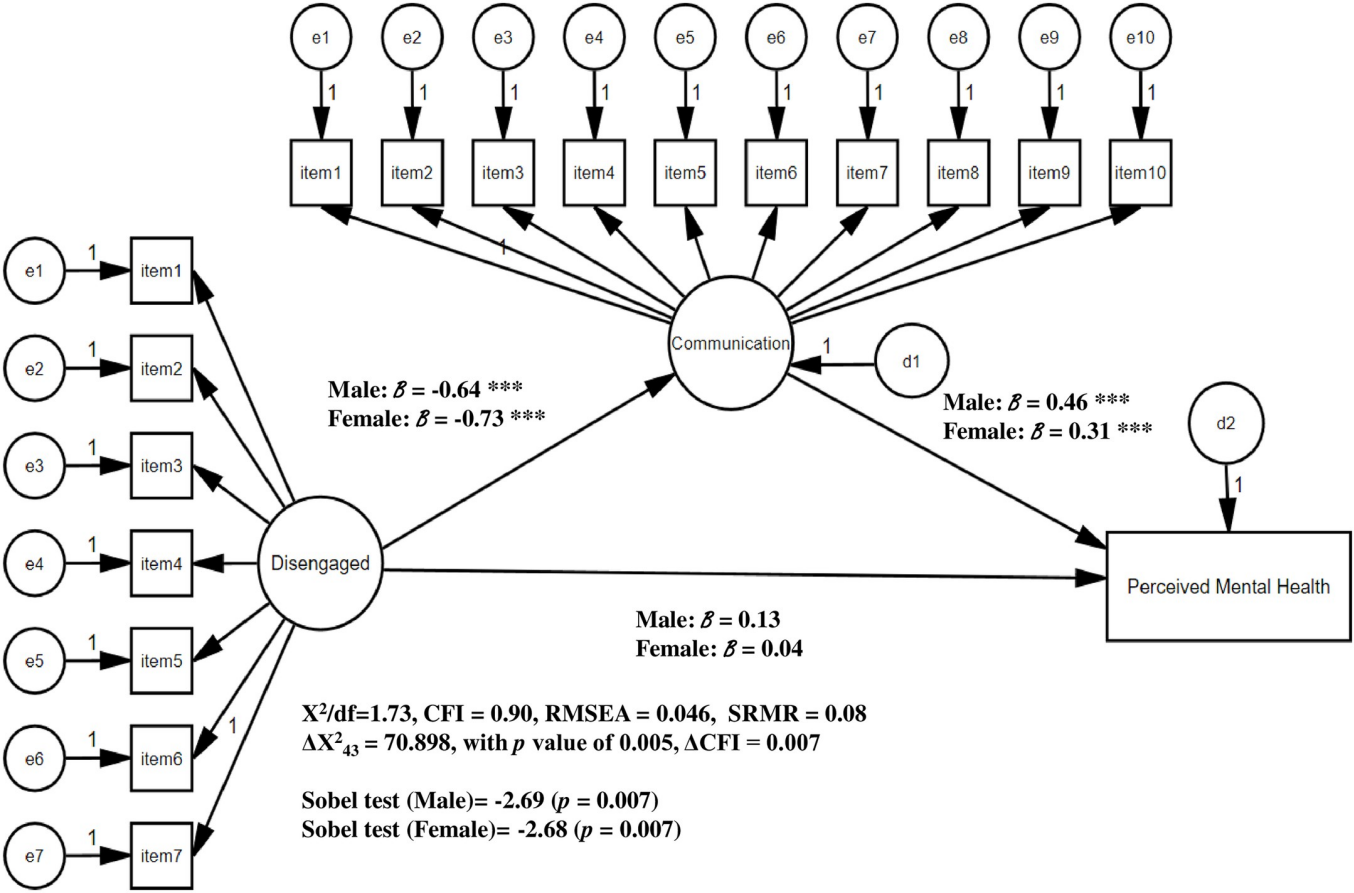
Family dysfunctions (disengaged), communication, and perceived mental health status: Moderating effect of gender (Model 1). *Note*. Socio-demographic factors (race, household annual income, school year, parents’ matiral status, parents’ educational attainment) were controlled. † *p* < .10, * *p* < .05, ** *p* < .01, *** *p* < .001.

**Fig 3:**
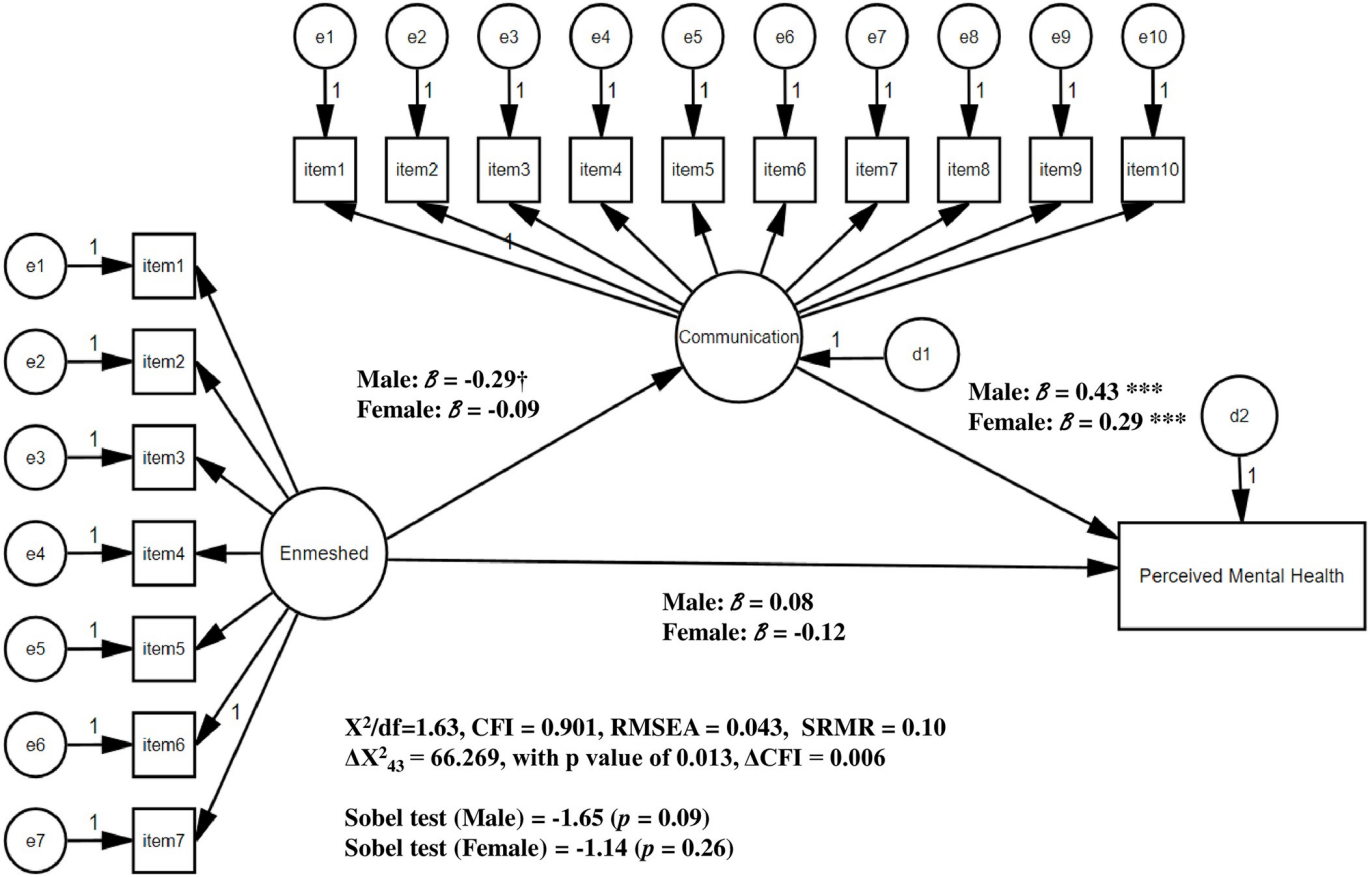
Family dysfunctions (enmeshed), communication, and perceived mental health status: Moderating effect of gender (Model 2). *Note*. Socio-demographic factors (race, household annual income, school year, parents’ matiral status, parents’ educational attainment) were controlled. † *p* < .10, * *p* < .05, ** *p* < .01, *** *p* < .001.

For the hypothesis 2 (the model comparison test), initially, there were no significant differences observed in the constrained measurement weights: (1) the model on the relationships among disengaged function, family communication, and perceived mental health reported that ΔX^2^_17_ = 21.771, with p value of 0.194 and ΔCFI = 0.001, and (2) the model on the relationships among enmeshed function, family communication, and perceived mental health showed that ΔX^2^_17_ = 23.611, with p value of 0.130 and ΔCFI = 0.002. These results indicate that items of family dysfunctions (disengaged and enmeshed) and family communication significantly reflect the constructs. Furthermore, there was no difference observed across the genders, which allowed us to evaluate the models with constrained structural weights for the next step. As the Figs 2 and 3 present, model comparisons of the structural weights constrained reported significant differences in the models 1 and 2: (1) the model on the relationships among disengaged function (Fig 2: model 1), family communication, and perceived mental health showed that ΔX^2^_43_ = 70.898, with *p* value of 0.005 and ΔCFI = 0.007 (2) the model on the relationships among enmeshed function (Fig 3: model 2), family communication, and perceived mental health indicated that ΔX^2^_43_ = 66.269, with *p* value of 0.013 and ΔCFI = 0.006. Details are as follows: First, the Sobel test confirmed the mediation effects of family communication on the relationships between disengaged functions and perceived mental health status for both groups (Sobel test = -2.69, -2.68; *p* = 0.007 for both males and females, respectively). Comparing the magnitude of the standardized B of this model, the effect of disengaged function on family communication of females (B = -0.73) was higher than males (B = -0.64), while the effect of communication on perceived mental health status of females (B = 0.31) was lower than males (B = 0.46). However, for the control variables (Fig 2, model 1), male students with married parents showed a higher level of perceived mental health status (B = 0.796, *p* = 0.004). Next, the Sobel test did not confirmed the mediation effects of family communication on the relationships between enmeshed functions and perceived mental health status (Fig 3, model 2; Sobel test = -1.65, -1.14; *p* = 0.09, 0.26 for males and females, respectively). For both groups, enmeshed dysfunctions did not significantly influence family communication, while communication significantly impacted perceived mental health status (B = 0.403, 0.287; *p* < .001 for both males and females, respectively). However, for the control variables (Fig 3, model 2), male students with married parents showed a higher level of perceived mental health status (B = 0.28, *p* = 0.006).

## Discussions

The overarching goals of this study were to investigate whether family communication mediates the relationships between family dysfunctions (disengaged or enmeshed) and perceived mental health status among undergraduate and graduate students. Additionally, the study aimed to explicate whether these pathways vary by gender.

Initially, although there was no significant direct impact observed from disengaged functions on perceived mental health status, the mediation role of family communication was identified in the pathway ’disengaged—family communication—perceived mental health status.’ This finding emphasizes the significant impact of family communication on undergraduate and graduate students. Even if families struggle with poor functioning, like not being very connected, when they use effective communication methods, these student groups experience better mental stability.

The lack of a significant direct effect of disengaged functions on perceived mental health status was inconsistent with findings from certain prior studies. For example, Freed et al. (2016) [10] reported a direct relationship between family functioning and children’s levels of depressive symptoms. McLeod, Weisz, and Wood (2007) [51] and Sheeber, Hops and Davis (2001) [52] also found that family functioning and interpersonal family factors consistently remained significant factors in the context of depressive symptoms in children. There are several potential reasons for the difference in the current study findings and prior research: (1) First, we could consider the residential status of the target groups. As none of the target groups were living with their parents at the time of data collection, their current residential status could influence their response patterns (the target participants were living away from their parental homes [e.g., dormitories or off-campus accommodations]). The findings of the current study indicate that, since most students are physically separated from their families during the semester, the perceived distance or connection status with family members did not critically influence the capture of the definition of the concept of family disengagement. (2) Next, the participants’ developmental transition status should also be considered. According to Arnett (2004) [53], emerging adulthood has self-focused characteristics. Most American emerging adults move away from their parental homes for various reasons [53] and establish a more independent lifestyle than they had as adolescents [54]. In other words, during the emerging adulthood period, individuals tend to focus more on their own independent lives and come to accept the necessity of learning to make decisions independently. Although Arnett (2000) [4] previously suggested that the onset of newfound independence during the emerging adulthood period could contribute to psychological distress, he concludes that psychological distress during emerging adulthood is impacted by complicated and diverse factors. The existing family boundaries might not be the sole cause of severe psychological distress among the target groups in the current study.

Hence, it is crucial to focus on the mediating role of family communication between family dysfunctions and the perceived mental health status of undergraduate and graduate students. For undergraduate and graduate students, family communication is a crucial factor for their mental health, and this communication can be influenced by family dynamics (i.e., indirect effect of disengagement on perceived mental health status). According to Olson (2011) [15], higher levels of family communication are related to their healthy family functioning (as opposed to the dysfunctional aspects seen in disengaged or enmeshed patterns). Previous research reinforces this outcome in that families with imbalanced family functioning often adopt negative or problematic interactional patterns such as poor family communication [15, 19]. While disengaged family boundaries could impact family communication strategies, members who maintain positive communication patterns within their family units are more likely to feel safe and comfortable. Family communication patterns or strategies manifest as visible and tangible behavioral outcomes, leading family members to evaluate their family dynamics based on these concrete patterns rather than abstract concepts like disengaged family boundaries.

In the context of enmeshed family functioning, the full model indicated that while enmeshed functioning was not significantly related to family communication, communication was significantly related to the perceived mental health status of the target groups. The scale reflecting enmeshed functioning highlights the importance of spending more time with family members. The enmeshed construct underscores that engaging in numerous activities with family members corresponds to a higher enmeshed score, indicating a greater level of attachment (unhealthy attachment). The current findings demonstrate that spending more time engaging in many activities cannot determine family communication patterns. This reflects that healthy relationships (quality of relationships) among family members are more important than the quantity of activities they engage in. The quality of interactions between family members can be determined by their communication with each other. Positive communication can enhance mutual respect and contribute to overall family mental stability. While frequent engagement in activities with family members can be enjoyable, if positive communication strategies are not maintained within family units, members might not feel mentally comfortable.

Regarding gender differences in the relationships among dysfunctions, communication, and mental health status, disengaged family functioning notably affects family communication and subsequently influences perceived mental health status for both males and females. No significant variations were found in these pathways across genders.

Specifically, both groups showed mediation effects of the communication between disengaged and perceived mental health status;. Johnson et al. (2010) [3] reported findings similar to those of the current study: there were no gender differences in family environment, including family communication patterns, emotional relationships, or life adjustment patterns. Raudino, Fergusson, and Horwood (2013) [55] also found no difference in the relationships between the quality of parent-child interactions and children’s psychological well-being for male and female groups. The lack of significant difference between females and males in the current study may be attributed to the item contents derived from the FACES IV scale, which reflect the family communication construct. The family communication scale includes balanced items for both emotional and problem-solving contexts [15]. Instead of capturing specific forms of family communication, such as emotional sensitivity or objective focus, which might cause differences between males and females, the given measurement concentrates on assessing how family members establish a balanced communication structure within the family. As Fig 3 shows, the model 2 (the path model involving enmeshed dysfunction, family communication, and perceived mental health status) exhibited consistent pattern with the model 1 in the gender difference analysis. The non-significant gender differences for the given paths could be attributed to the sample size of the current study. The study does not have equal gender variability within the total sample size. While this might not be a critical factor for the research findings, we should consider the potential influence of sample size.

However, marital status of parents (one of the control variables) showed significant difference between males and females (for both Models), and this finding consistent with the prior study findings [56]. The authors found that female groups were more likely to be influenced by actual relationships with family members in the family units, while male groups were more likely to be influenced by structural and growth factors, such as the marital status of parents.

Except for the marital status of the male group, there was no significant influence of the controlled socio-demographic factors such as race, household income, school year, and parents’ educational attainment.

### Strengths and limitations

The current study is particularly meaningful as it examines how specific family dysfunctions, such as disengagement and enmeshment, influence family communication and perceived mental health. Furthermore, the study collected original data targeting undergraduate and graduate students. It is significant to discover that positive family communication is one of the most crucial factors in family dynamics, highlighting the realistic and tangible nature of family interactions. This will help guide practitioners in developing and disseminating appropriate intervention programs for community members.

There are several limitations of the current study. The participants were composed of both undergraduate and graduate students from Ohio in the United States, so the findings might not be representative of all undergraduate or graduate students across the U.S. The use of convenience sampling could restrict the applicability of the results, comparing to the random sampling. Concerning the sample size of each group (male and female), 73.6% were female and 26.4% were male, indicating unequal distribution between the two groups. Differences in group size could affect the group differences observed when conducting multiple group analysis in the current study. In addition, providing more specific outcome values could help us identify which factors are more likely to be related to students’ mental health status. The current study employed straightforward questions to assess perceived mental health status. Utilizing more specific categories, such as self-esteem, anxiety, depression, or happiness, could lead to a more comprehensive understanding of the relationship between family communication and mental health status among undergraduate students.

With regard to above limitations, future studies could consider appropriate gender variability within the target group to enhance the generalizability of the study. Additionally, employing more specific and detailed scale-level measures would be necessary for capturing the definition of perceived mental health. Furthermore, the current study relies on a cross-sectional research design. Future research could utilize a longitudinal research design to capture more specified causal relationships across family dysfunctions, communication patterns, and perceived mental health status. Future research should focus on dyadic relationships between parents and emerging adult children at multiple time points. For instance, researchers could examine the relationships among family dynamics, communication, and mental health during the freshman year, followed by additional assessments at multiple time points leading up to graduation. As the current study found the critical role of family communication in family units, future research could help in developing ‘intervention programs’ to enhance positive family communication strategies for community members. According to Minuchin (2007) [31], asking following questions would be helpful in understanding the condition of family units (whether they are healthy or unhealthy) [32]: ’What are the family’s patterns of interacting?’ ’How does the family present itself structurally?’ ’Where does the power lie in this family?’ ’In what contexts?’ These questions support the construct of ‘family members’ relationship quality. In line with Minuchin’s (2007) guidelines, practitioners could design appropriate assessment tools for the target families and provide effective intervention sessions.

## Conclusions

The current study revealed that a balanced family structure, characterized by appropriate family boundaries, promotes the ability of undergraduate and graduate students to communicate effectively with family members [33] and maintain better perceived mental health status. Most importantly, improved family communication has the crucial power to positively influence the perceived mental health status of under/graduate students. Practitioners should recognize that improving the positive communication skills within the family unit can be a realistic approach to support under/graduate students’ perceived mental health status. Colleges and universities should offer regular education programs to promote understanding of the relationship between family communication strategies and the mental health status of undergraduate and graduate students. According to Minuchin [32], the following strategies could be implemented in the department of student counseling on campus or in family centers in communities: (1) teaching communication skills such as speaking, listening skills, and managing conflicts, (2) assigning tasks for implementation in the nature environment, and (3) performing role-plays or role reversal strategies. Additionally, to enhance the quality of family communication, practitioners can assess the communication strategies used in family units, including blaming, placating, computing, distracting, and leveling strategies [57]. Then, practitioners can guide family members to adapt to a new or different communication style if their current behavior patterns are not appropriate.
